# Longitudinal imaging of the taste bud *in vivo* with two-photon laser scanning microscopy

**DOI:** 10.1371/journal.pone.0309366

**Published:** 2024-12-13

**Authors:** Brittany N. Walters, Zachary D. Whiddon, Aaron W. McGee, Robin F. Krimm

**Affiliations:** 1 Department of Anatomical Sciences and Neurobiology, University of Louisville School of Medicine, Louisville, KY, United States of America; 2 Department of Neurobiology, University of California San Diego, La Jolla, California, United States of America; Wrocław University of Environmental and Life Sciences: Uniwersytet Przyrodniczy we Wroclawiu, POLAND

## Abstract

Taste bud cells in the tongue transduce taste information from chemicals in food and transmit this information to gustatory neurons in the geniculate ganglion that innervate taste buds. The peripheral taste system is a dynamic environment where taste bud cells are continuously replaced, but further understanding of this phenomenon has been limited by the inability to directly observe this process. To overcome this challenge, we combined chronic *in vivo* two-photon laser scanning microscopy with genetic labeling of gustatory neurons and taste buds to observe how cells within the taste bud change over time. This method expands the investigative possibilities beyond those offered by fixed-tissue methods. This method permits direct observation of taste bud cell entry, cell differentiation, cell loss, and arbor plasticity. We demonstrate that a few stains/dyes can be used to observe nuclei and organelles in the taste bud *in vivo*. We also describe a workflow for reconstructing composite z-stacks with grayscale data of both cells and arbors using ImageJ, Neurolucida 360, and Neurolucida Explorer software. Together, the methodology and software options for analyses presented here provide a novel approach for longitudinally observing taste bud cells and arbors in the taste bud *in vivo*.

## Introduction

Cells in taste buds are continuously renewed [1–4]. However, the processes, cellular lifespans, and dynamic connectivity in this structure are poorly understood. Taste buds are composed of 50–100 columnar epithelial cells, which are classified into three taste bud cell types based on their morphology and stimuli transduced [1–4]. Type I taste bud cells have cytoplasmic extensions that wrap around other taste bud cells and are thought to primarily have glia-like functions. Type II taste bud cells generally transduce bitter, sweet, and umami stimuli [3, 5–7]. Type III taste bud cells generally transduce sour [3, 8–10], and salty (sodium chloride) stimuli; however, salty stimuli can be transduced by all three taste cell types [3, 11]. Taste bud cells arise from a progenitor cell population [12–14]. In response to signaling and transcription factors (i.e. Sonic Hedgehog (SHH) and SRY-Box Transcription Factor 2 (SOX2)) progenitors continue developing as post-mitotic precursor cells that have the capacity to differentiate into all mature taste cell types [12–14]. Studies of taste bud cells estimate that their lifespan is 10 days on average [12]; however, when lifespan was analyzed by cell type, type II and III taste bud cells were found to have half-lives of 8 and 22 days, respectively [15]. This difference in taste cell lifespans underscores the complexity of maintaining a sufficient population of each cell type to properly transduce taste information from chemicals in food to gustatory neurons without any appreciative difference in taste quality over time.

Individual gustatory neurons have a single peripheral axon that enters the tongue and, depending on the amount of axon branching, terminates in 1–14 separate arbors (the portion of the axon that branches as it innervates tissue) that form connections with taste bud cells. There can be more than one arbor in a single taste bud and most gustatory arbors have multiple branches [16]. Despite extensive research with fixed tissue that has dominated investigation of the taste system [3, 12, 15, 17, 18], questions persist regarding the dynamic nature of cell turnover and its impact on the morphology of arbors. Although these studies provide a general and static understanding of cellular phenomena, they fail to reveal the cellular movements of taste bud cells, arbors, and connections between the two structures. Other attempts to construct *ex vivo* systems that recapitulate the dynamics of the taste system have been developed but may produce alterations in cellular behavior due to the isolation of cells from their native environment [19, 20]. *In vivo* studies of the tongue aiming to capture this environment have been technically limited by difficulty removing and stabilizing the tongue in a consistent manner for repeated observation, and by the need for increased imaging depth to observe taste buds in the epithelium [21–24].

Here, we describe a method for visualization of the dynamic activity of taste bud cells and arbors *in vivo* with two-photon microscopy. Recent studies by Whiddon et al. (2023) and Wood et al. (2024) employed this method to evaluate arbor plasticity and the effects of chemotherapeutic agents on innervation within taste buds, respectively [25, 26].

## Materials and methods

Details of the protocol described here are also published on protocols.io DOI: dx.doi.org/10.17504/protocols.io.eq2lyjqoelx9/v1 and is included for printing purposes as S1 File.

### Animals

All animals were cared for in accordance with guidelines set by the U.S. Public Health Service Policy on the Humane Care and Use of Laboratory Animals and the NIH Guidelines for the Care and Use of Laboratory Animals. Approval number: 22151. All animals were anesthetized with isoflurane and euthanized with CO_2_ followed by cervical dislocation.

*TrkB*^CreER^ mice (*Ntrk2*^*tm3*.*1(cre/ERT2)Ddg*^; ISMR catalog #JAX:027214, RRID:IMSR_JAX:027214; [1, 27]) were crossed with Cre-dependent tdTomato mice (Ai14;RRID: IMSR_JAX:007914) to obtain *TrkB*^CreER^-tdTomato mice in which tdTomato is expressed following *TrkB-*driven Cre-mediated gene recombination. Because sensory neurons innervating fungiform taste buds express *TrkB* (Tropomyosin receptor kinase B) [27], it is possible to label arbors innervating taste buds by expressing tdTomato in this neuron population. These mice will be referred to as *TrkB-*tdTomato mice. These mice were crossed with a *Sox2*^GFP^ line (Sox2^tm2Hoch^; ISMR catalog #JAX:017592, RRID:IMSR_JAX:017592; [28]) to obtain *Sox2*^GFP^:*TrkB*-tdTomato mice. *KRT14*^CreER^ mice were crossed with Cre-dependent tdTomato mice to obtain *KRT14*^CreER^:tdTomato mice in which tdTomato is expressed following *KRT14*-driven Cre-mediated gene recombination. *Tas1r3-GFP* mice (a gift from Dr. Robert Margolskee [29]) were used to observe Type II taste bud cells and will be referred to as *T1R3*^GFP^ mice.

### Tamoxifen injections

Tamoxifen was used to induce gene recombination in inducible mouse lines. Tamoxifen was dissolved in corn oil at 20 mg/mL by shaking and heating at 42°C. On postnatal day 40, 1.5–4.0 mg of tamoxifen was injected via intragastric gavage. For the initial imaging session, male and female mice were used at postnatal day 60 or older. For best results, tamoxifen should be administered at least 4 weeks prior to imaging to sparsely label arbors.

### Application of stains and dyes to the tongue

For application onto the tongue, Hoechst 33342 was reconstituted to a concentration of 400 μg/mL in 1x phosphate-buffered saline (PBS). A 50μL aliquot of MitoView 405 was diluted in 75 μL of dimethyl sulfoxide and 400μL of 1x PBS (21.05 μM). Prior to application of the stain/dye, the mouse was anesthetized, and the tongue was withdrawn using plastic blunt-ended forceps. Using a different pair of blunt-ended forceps, the stain/dye was spread over the tongue and regularly reapplied at 5-min intervals for 20 min. Within 3 h of application—as suggested in [30]–Hoechst 33342 stain had labeled the nuclei of cells within the lingual epithelium. Following hourly evaluation of tissue post-application of MitoView 405, mitochondria were observed to be extensively labeled at 6 h.

### Intravital imaging

**Fig 1:**
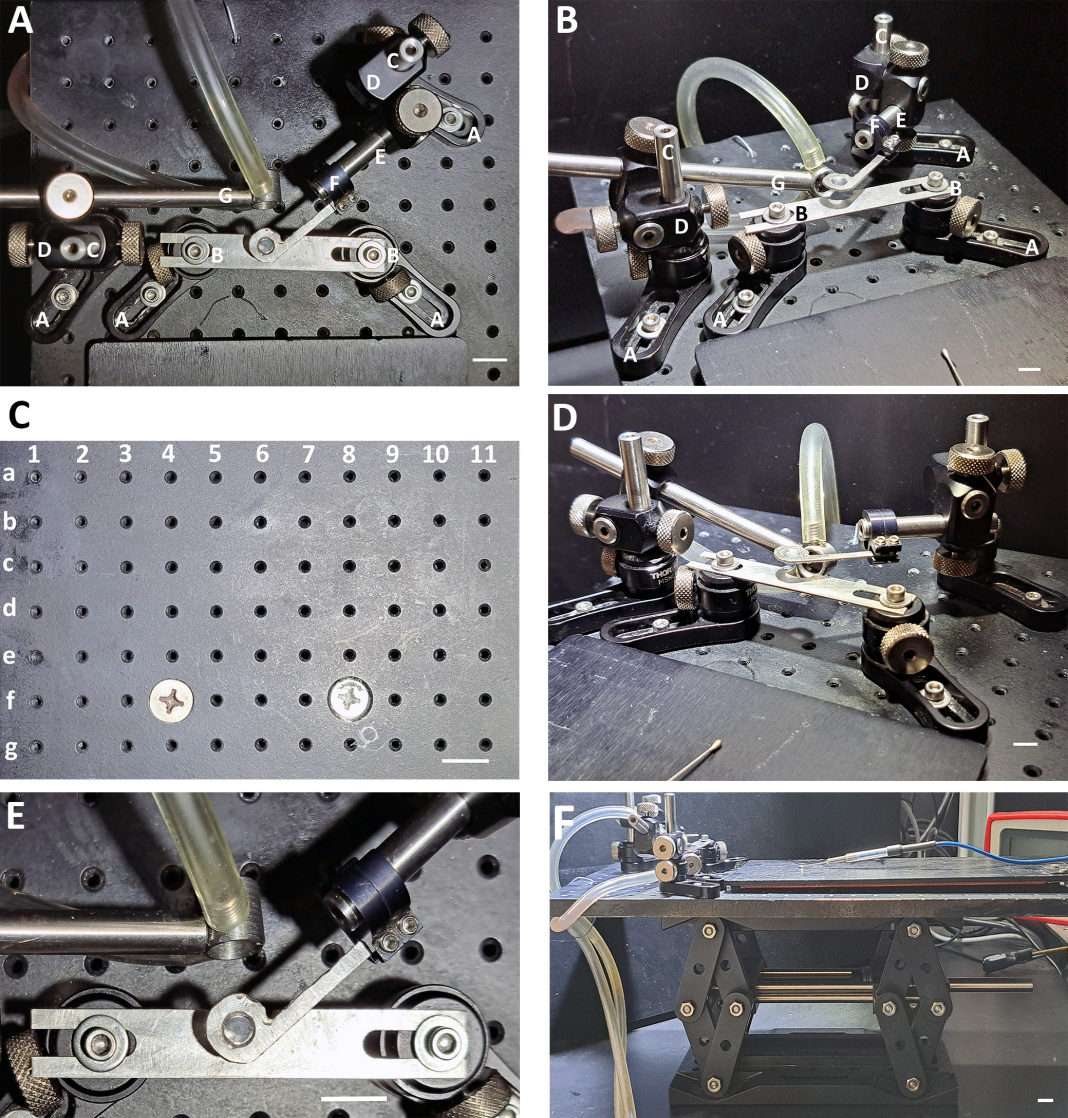
Constructing a custom-built imaging platform. (**A**) Top-down view of the stage component of the imaging platform with letters corresponding to the names of required parts and indicating the general order of stage assembly (e.g., steps A-G). (**B**) View of the stage from the left. (**C**) The stage is assembled by following the presented row-column (i.e., letter-number) schematic. This top-down view of the breadboard illustrates where specific parts should be positioned to simplify the assembly process. For step-by-step instructions on the assembly process, refer to S1 File. (**D**) View of the stage from the right. (**E**) Enlarged view of the cover glass holder and metal plate. (**F**) Lateral view of the stage, breadboard, and jack. Scale bar for A, B, and D-F: 1 cm; scale bar for C: 13 mm.

Repeated observation of taste buds is dependent on adequate initial tongue positioning and subsequent stabilization throughout the imaging session. This was achieved by assembling a custom-built imaging platform and cover glass holder (Fig 1). The required equipment for performing two-photon imaging, supporting ScanImage Software [31], and information on platform parts and assembly are provided here: DOI: dx.doi.org/10.17504/protocols.io.eq2lyjqoelx9/v1.

#### Positioning the tongue under the cover glass holder

**Fig 2:**
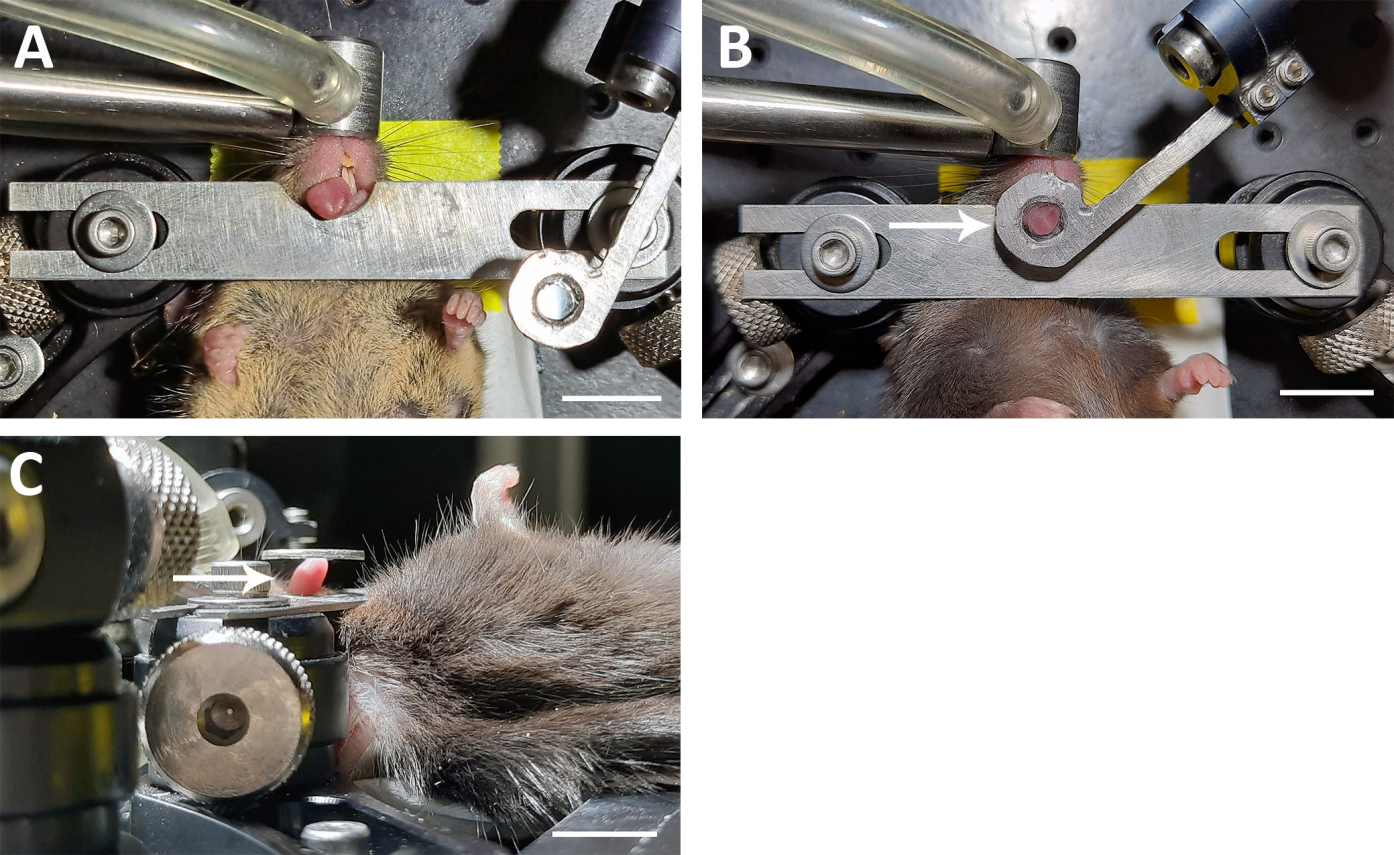
Preparing the mouse for imaging. (**A**) An anesthetized mouse on the imaging platform after the tongue had been withdrawn. (**B**) View from A but with the cover glass holder lightly depressing the tip of the tongue. (**C**) Lateral view of the mouse fixed in position with the cover glass holder depressed an appropriate depth for imaging. In panels B and C, white arrows indicate the location in which the temporary spacer can be inserted to set the distance between the cover glass holder and the metal plate. Scale bar for A-C: 1 cm.

A mouse was anesthetized with 5.0% isoflurane in an anesthesia induction chamber and placed in the supine position on the imaging platform. The nose was placed in the nosecone, and the percentage of isoflurane was reduced to ~1.5%. The tip of the tongue was carefully withdrawn using plastic blunt-ended forceps (Fig 2A). The design of the imaging platform was based on a similar, previously described design [22, 25]. However, we added the cover glass holder to include a coverslip which depresses the tongue just enough so that the surface under the coverslip is within the same focal plane. When lowering the cover glass holder, it can be useful to have a 1 mm or thicker rigid spacer next to the tongue and between the metal plate and cover glass holder (Fig 2B white arrows indicate location of spacer). This ensures a minimum distance is maintained between the metal plate beneath the tongue and the coverslip on top of the tongue. It is critical that the cover glass holder not compress the tongue but only flatten the tip as depicted (Fig 2C). The imaging platform was moved under the Moveable Objective Microscope (MOM) (Sutter Instruments), and drops of distilled water were placed on top of the coverslip.

#### Imaging papillae maps

**Fig 3:**
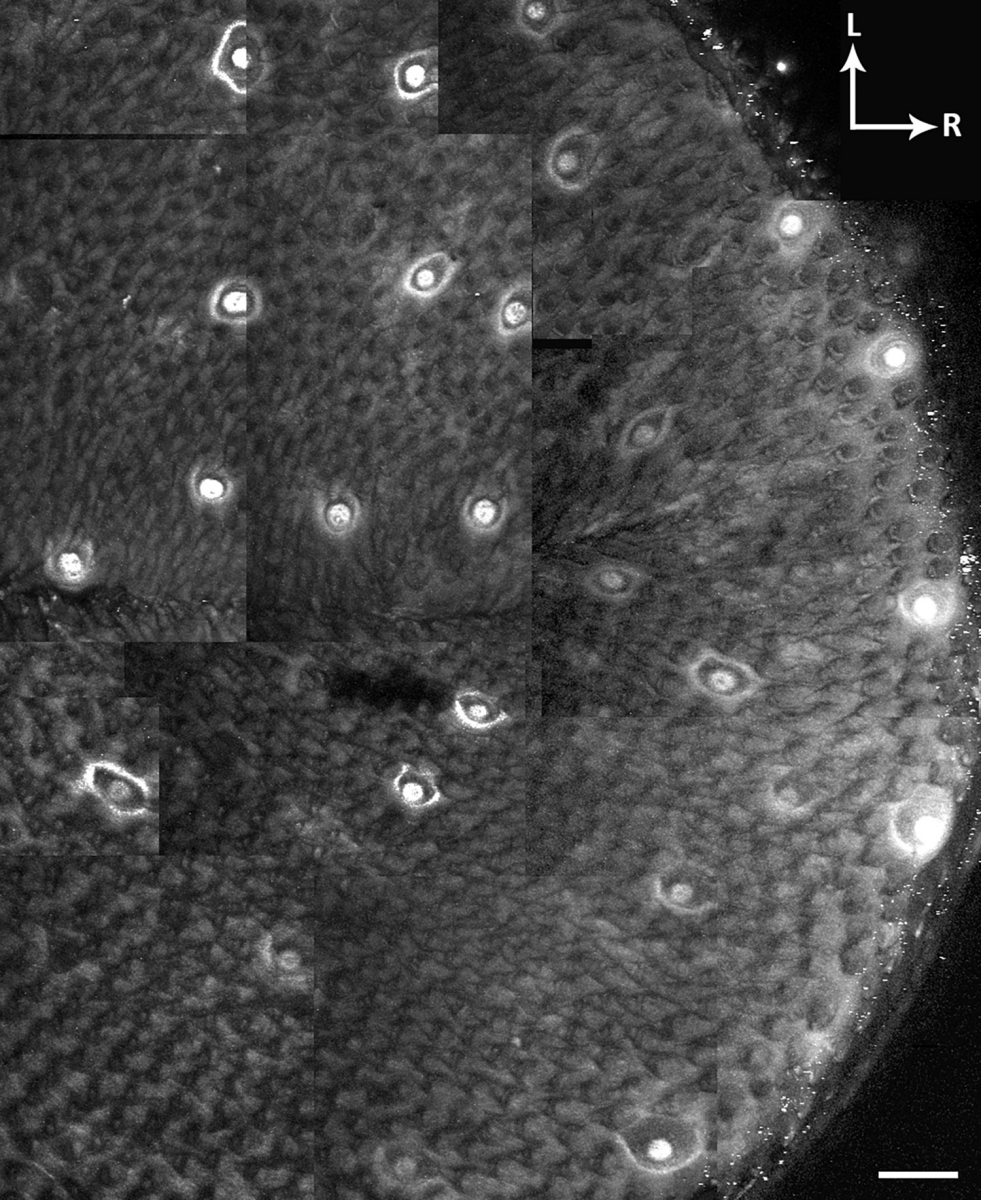
Generating a papillae map. Papillae map constructed of multiple independent composite z-stacks showing *Sox2*^GFP+^ fungiform taste buds on the surface of the anterior tongue of a *Sox2*^GFP^ mouse. ‘L’ indicates lateral, and ‘R’ indicates rostral. Scale bar: 100μm.

A 10X 0.3 numerical aperture (NA) W N-ACHROPLAN (Zeiss) objective and MOM were used for imaging. The objective was positioned over the epithelium of the anterior tongue. A z-stack spanning from the basal lamina to the dorsal epithelium was acquired for the left and right halves of the tongue. The composite z-stack for each papillae map was reconstructed using ImageJ software. The two papillae maps were adjoined at the tongue midline to prevent duplicate imaging of taste buds (Fig 3). To help identify the same taste buds during subsequent imaging sessions, a unique feature on the papillae map was noted (e.g., close proximity of two taste buds to each other relative to other taste buds, particular angle of two or three taste buds on the tongue). Identifying this feature on the map with the 10X objective facilitates its subsequent identification under a 40X 1.0 NA W Plan-Apochromat objective (Zeiss).

**Fig 4:**
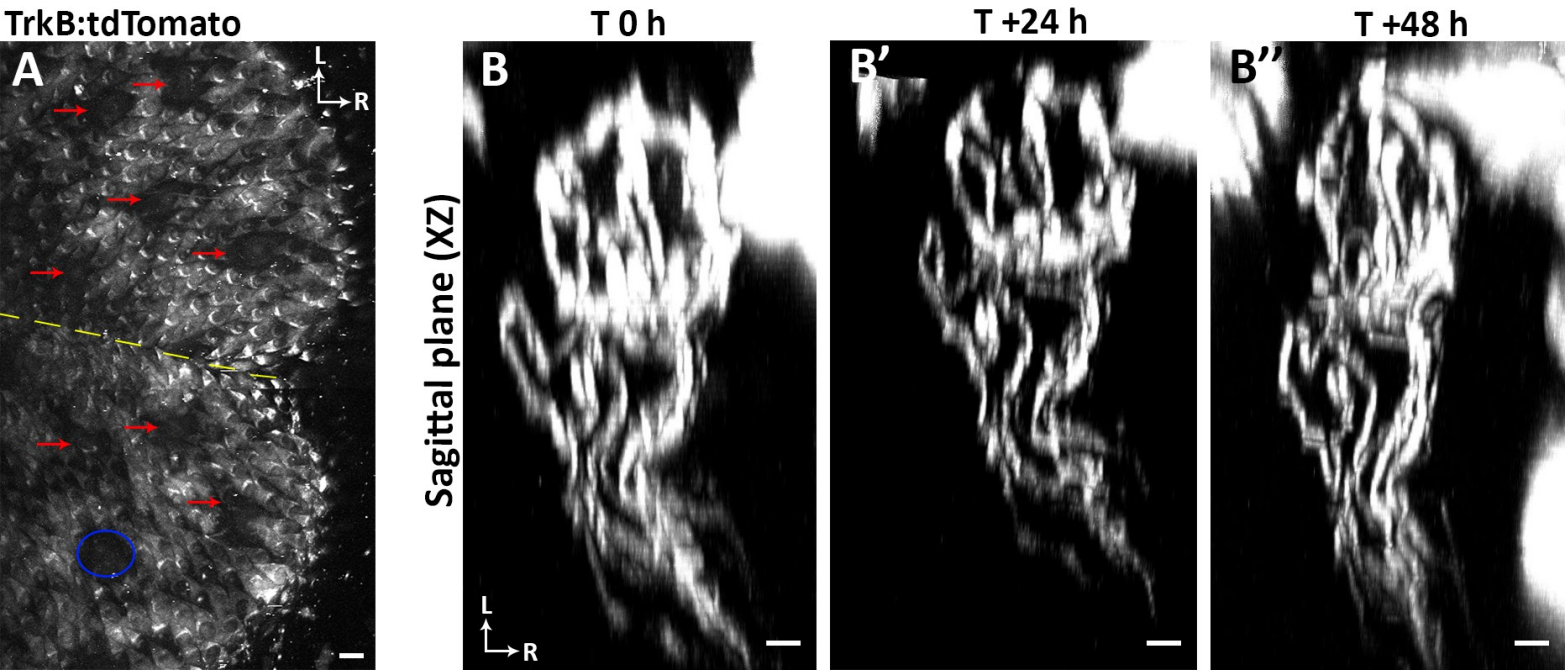
Tracking the same taste buds in *TrkB*-tdTomato mice using only autofluorescence. (**A**) A papillae map from a *TrkB*-tdTomato mouse without *Sox2*^GFP^ labeling in fungiform taste buds. In this case fungiform papillae (red arrows) can be identified by the absence of filiform autofluorescence. Filiform papillae tend to have autofluorescence perhaps because of their heavy keratinization. The yellow dashed line depicts the location of tongue midline, and the blue ellipse indicates the location of the fungiform papilla and arbor shown in panels B-B”. (**B-B”**) Multiple terminal arbors were labeled in each taste bud following administration of 4.0 mg tamoxifen. Labeled innervation to a single taste bud is shown from the sagittal plane (XZ) at 0, 24, and 48 h. The arbors can be represented in 3D by first processing the composite z-stack in ImageJ (refer to Steps 9.1–9.3 in S1 File) and then importing it into Neurolucida 360.For panels A and B-B”, ‘L’ indicates lateral, and ‘R’ indicates rostral. Scale bar for A: 30μm; scale bar for B-B”: 5μm.

**Fig 5:**
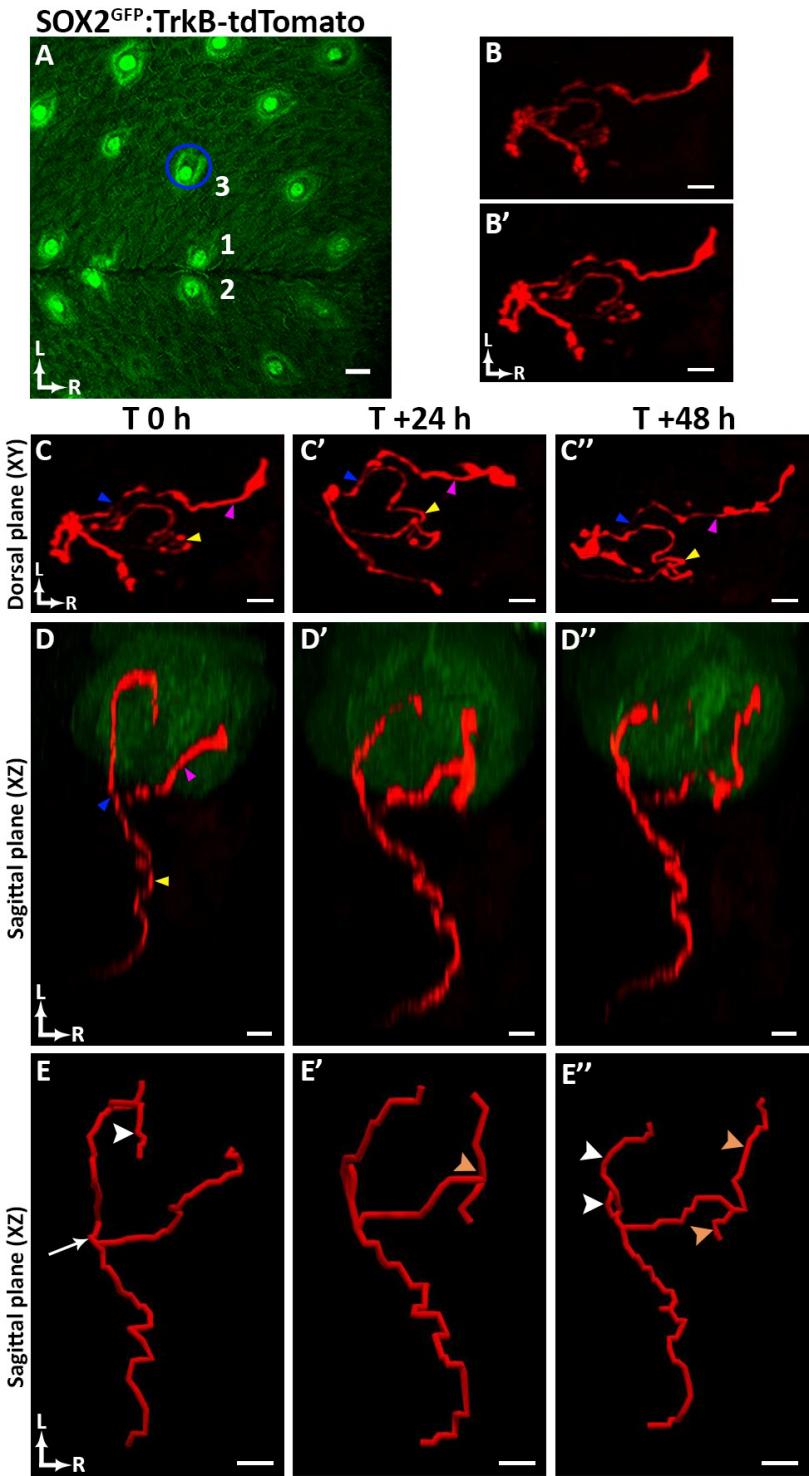
Tracking arbor plasticity in *Sox2*^GFP^:*TrkB-*tdTomato mice. (**A**) This papillae map was created from a *Sox2*^GFP^:*TrkB-*tdTomato mouse. The blue ellipse indicates the location of the taste bud and arbor represented in panels B-B’, C-C” and D-D”. Example of an arbor prior to deconvolution (**B**), and the same arbor after deconvolution using AutoQuant X3 software (**B’**). (**C-C”**) 3D dorsal plane (XY) of each terminal arbor (pseudo-colored red). Each arbor may appear more complicated because parts of the arbor were obscured in panels B-B” by the taste bud, which is not included here for simplicity. (**D-D”**) Sagittal plane (XZ) of the taste bud (pseudo-colored green) and the terminal arbors (pseudo-colored red) at 0, 24, and 48 h. The blue arrowhead indicates the bifurcation of the arbor prior to entering the taste bud, the pink arrowhead indicates one of two major branches from the branch point at the base of the taste bud, and the yellow arrowhead indicates the portion of the arbor beneath the taste bud. (**E-E”**) Arbor tracings from T 0 to T + 48 h. As seen from the tracing, this arbor had one bifurcation as it entered the taste bud that remained constant across imaging (panel E, white arrow), while terminal arbors remodeled within the 48-h period. From T0 to T + 24 h, one of the three terminal branches on the rostral side of the arbor retracted (panel E, white arrowhead), and there was growth of a terminal branch on the caudal side of the arbor (panel E’, orange arrowhead). From T + 24 to T + 48 h, the secondary branches on the rostral side of the arbor shortened (panel E”, white arrowheads), whereas the secondary branches on the caudal side of the arbor increased in length (panel E”, orange arrowheads indicate branch growth and white arrowheads indicate branch retraction). For all panels, ‘L’ indicates lateral, and ‘R’ indicates rostral. Scale bar for A: 30μm; scale bar for B-B’ and C-C”: 3μm; scale bar for D-D” and E-E”: 5μm.

**Imaging taste buds.** The tongue of each mouse has a unique taste bud pattern which is enhanced by GFP expression in the anterior tongue epithelium. This pattern of taste buds is readily visible with a 10X lens and provides a set of fiduciary points to repeatedly locate the same taste buds. To best image individual taste buds, the 10X lens needs to be replaced with a 40X lens. When a taste bud is positively identified on the papillae map, a z-stack is created spanning from the base of the taste bud—or when arbors were no longer visible—to the taste pore (Figs 4 and 5) by imaging every 1μm in the z-axis. Green fluorescent protein (GFP) was visualized with a Chameleon Ti:Sapphire mode-locked, tunable laser (Coherent) set to 920 nm, and tdTomato was visualized with a Fidelity-2 1070 nm laser (Coherent, Silicon Valley). Z-stacks were collected with power set to 2.75 W. The excitation filters used were the 612/69 nm BrightLine® single-band bandpass filter to selectively filter signals for GFP, and 510/844 nm BrightLine® single-band bandpass filter to selectively filter signals for tdTomato. The sampling interval was set to 1 optical section/second. Each taste bud is assigned a number after image collection which is placed at the corresponding location on the papillae map. This process can be repeated until the required number of taste buds are collected. Upon completion of imaging, the mouse is removed from the imaging platform, a drop of artificial saliva placed on its tongue, and it is returned to its home cage for recovery.

### Analysis workflow

Composite z-stacks with grayscale data of both cells and arbors were deinterleaved and merged to pseudo-color each channel (Fig 5). The composite tif stacks were deconvoluted using AutoQuant X3 and imported into Neurolucida 360 software to view the cells and trace the arbors in 3D.

## Results

### Papillae maps can be rendered via fluorophore signal or autofluorescence

Fungiform papillae form a unique pattern across the tongue that is stable over time [32, 33]. This hallmark feature can be exploited to construct papillae maps which can be used to repeatedly identify the same taste buds over time. The best maps were acquired using *Sox2*^GFP^ mice, which express GFP in fungiform taste buds, as the fluorescence signal associated with GFP facilitates the identification of fungiform papillae (Fig 3) [28]. However, it is possible to identify fungiform papillae without GFP by using the autofluorescence of filiform papillae to visualize the distribution of fungiform papillae as an array of dark spots (Fig 4A). Because GFP is no longer being used to generate a map, this approach would make it possible to use GFP as a second label in specific cell types or organelles.

### Tamoxifen dosage influences the amount of labelled innervation to the taste bud

Taste arbors can be visualized in *TrkB*-tdTomato mice that express tdTomato in subsets of arbors following injection with 1.5 mg tamoxifen [25]. Each individual taste bud could be readily resolved in a papillae map composed of multiple tiled z-stacks created from a *Sox2*^GFP^:*TrkB*-tdTomato mouse (Fig 3). A larger dose of tamoxifen (4.0 mg) was administered to this mouse, resulting in many labeled arbors to each taste bud. The higher the dosage of tamoxifen administered, the more neurons that undergo gene recombination, resulting in the appearance of more labeled arbors innervating each taste bud (Fig 4B–4B”). However, labeling too many arbors per taste bud limits the ability to reconstruct individual arbors using Neurolucida 360.

### Analyzing arbor variation following longitudinal imaging of the taste bud

Using the papillae map constructed from a *Sox2*^GFP^:*TrkB*-tdTomato mouse (Fig 5A), multiple taste buds were tracked across time, and one representative taste bud was selected to illustrate arbor plasticity. To best illustrate arbor structure, image stacks containing arbor data were deconvoluted using AutoQuant X3 software (Fig 5B–5B’). The specific arbor innervating this taste bud is represented in the dorsal plane (XY) (Fig 5C–5C”) as well as in the sagittal plane (XZ) at T0, T + 24, and T + 48 h (Fig 5D–5D”). To observe changes in arbor length over time, composite z-stacks acquired for each time point were imported into Neurolucida 360 for tracing. Following tracing, the length of the traced arbor and associated branches was measured using Neurolucida Explorer. Traced arbors are shown in the sagittal plane (XZ) at T0, T + 24, and T + 48 h (Fig 5E-5E”). When analyzing this arbor over time, the primary bifurcation of the arbor entering the taste bud remained constant, whereas the terminal branches remodeled. At 0 h, the arbor had two secondary branches, one of these did not branch further while the other had three terminal branches. However, 48 h later, both secondary branches remained, and each had two terminal branches. Thus, as previously described [25], plasticity of terminal branches occurs at a rapid pace.

### Using stains and dyes to visualize structures within the taste bud

**Fig 6:**
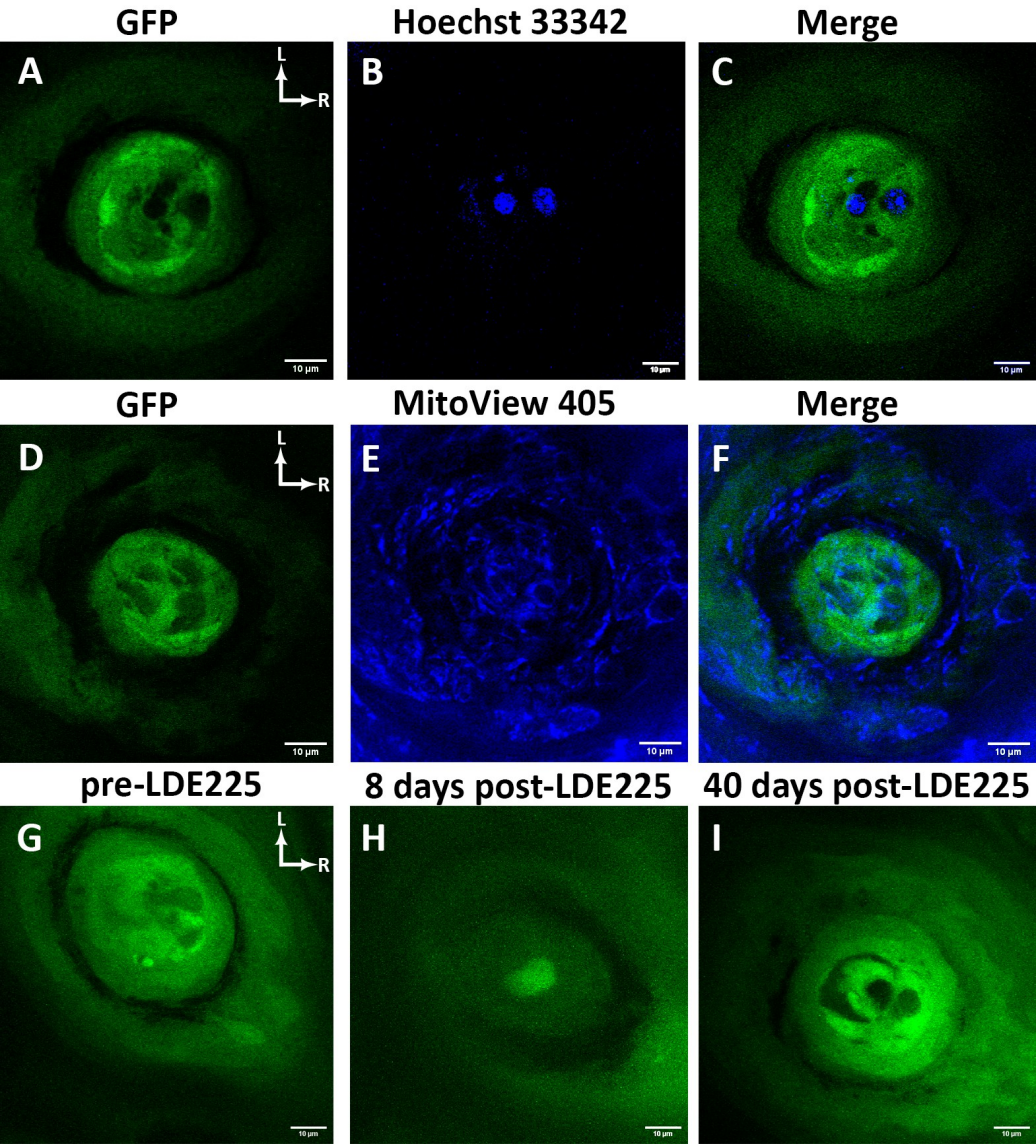
Applying stains and dyes to the tongue and oral administration of chemotherapeutic agents. (**A-C**) A horizontal section through a taste bud showing two cell nuclei labeled with the blue, fluorescent Hoechst 33342 stain in a *Sox2*^GFP^:*TrkB-*tdTomato mouse. Four optical sections from the z-stack were merged to best visualize the stain in taste bud cells. Hoechst 33342 also labeled the nuclei of keratinocytes (not shown). (**D-F**) A taste bud with mitochondria stained by the fluorescent mitochondrial dye MitoView 405 in a *Sox2*^GFP^ mouse. (**G-I**) A taste bud observed over time in a *Sox2*^GFP^ mouse following oral administration of the chemotherapeutic agent Sonidegib (LDE225). Representative images of LDE225-treated taste buds demonstrate (**G**) a morphologically normal taste bud prior to LDE225 treatment, (**H**) degeneration of the taste bud by day 8 of treatment, and (**I**) recovery of the taste bud by day 40 post-treatment. Rostral and lateral directions are indicated by white arrows in A-I. Scale bar for A-H: 10μm.

In addition to genetic markers, stains and dyes can be directly applied to the anterior tongue to visualize nuclei and organelles. We found that application of Hoechst 33342 to the tongue stained multiple cell nuclei in most taste buds in *Sox2*^GFP^ mice. Hoechst 33342 also labeled keratinocytes surrounding the taste bud, which obscured the visibility of individual cell nuclei. For this reason, a select few adjacent optical sections were combined with both the green (GFP label) and blue (Hoechst 33342 stain) to illustrate that nuclei were labeled in specific cells (Fig 6A–6C). Dyes can also be applied to the tongue surface to directly observe organelles in live cells. Application of the fluorescent mitochondrial dye MitoView 405 allowed real-time observation of mitochondria in taste bud cells in *Sox2*^GFP^ mice (Fig 6D–6F). Again, a few optical sections were selected to produce the merged image. This approach could be used to examine changes in organelles within taste bud cells as they mature and degenerate. The ability to combine genetic labels with stains provides further opportunities to address additional research questions.

### Observing the effect of chemotherapeutic agents on taste buds

Taste buds can be examined before and after drug treatment using this method. For example, chemotherapeutic agents such as Sonidegib (LDE225) can be administered via oral gavage to observe drug effects on the same taste buds over time [34]. When *Sox2*^GFP^ mice were treated with the chemotherapeutic agent LDE225, degeneration and regeneration of taste buds were observed over time. For example, a morphologically normal taste bud (Fig 6G) showed rapid degeneration by day 8 of LDE225 treatment (Fig 6H), yet by day 40 post-treatment, had recovered its characteristic morphologic features (Fig 6I).

### Observing different stages of the taste bud cell cycle using genetic labels

**Fig 7:**
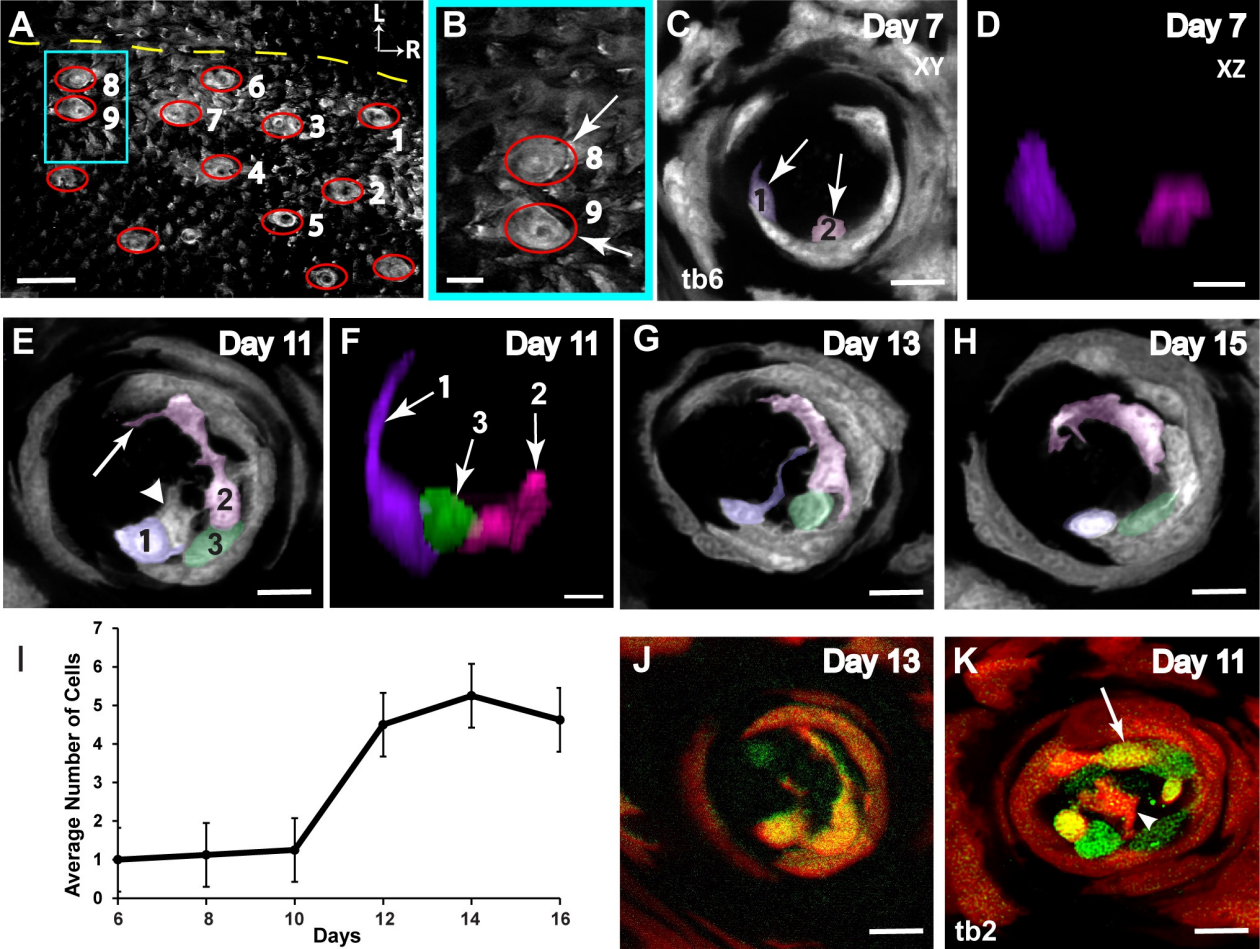
Examining cell entry into the taste bud. (**A**) The red ellipses denote fungiform papillae, and the yellow dashed line depicts the tongue midline in a *KRT14*^CreER^:tdTomato mouse. (**B**) Two immediately adjacent papillae (8 and 9) were used as a fiduciary point for each imaging session. (**C**) Taste buds were imaged every 48 h, and one representative taste bud (tb6) is shown. *KRT14*^+^ progenitors form a ring around the outside of the taste bud (white arrows), allowing the taste bud to be defined as the dark space in the middle with two cells inside the bud (pseudo-colored purple and pink). (**D**) The two cells inside the taste bud on day 7 (after tamoxifen injection on day 1) were segmented using Imaris. All fluorescence outside the cells was removed so that the cells could be viewed in the YZ plane. (**E**) By day 11, three pseudo-colored taste bud cells could be followed across the 5 days. (**F**) Segmenting the cells revealed that cells 2 and 3 had an immature morphology, whereas cell 1 had a mature morphology, identified by a process extending to the taste pore. (**G**) One precursor cell (between cells 1 and 3) was lost by day 13 and never matured (white arrowhead), (**H**) but all other cells remained in the taste bud through day 15. (**I**) The *KRT14*^CreER^:tdTomato mice were injected with tamoxifen on Day 0 and subsequently imaged every 48-h. From days 6 to 12, taste buds showed an increase in the average number of new tdTomato+ cells, with a peak occurring on day 14. Standard error bars are shown. (**J**) All three cells inside tb6 were *T1R3*^GFP+^; (**K**) however, taste bud 2 (tb2) had both GFP^+^ (white arrow) and GFP^-^ (white arrowhead) tdTomato-labeled cells. Scale bar for A: 200μm; scale bar for B: 100μm; scale bar for C, E, G, H, J, and K: 10μm; scale bar for D and F: 3μm.

Furthermore, genetic labels can be used to observe cell differentiation in taste buds. For example, a single 1.0 mg dose of tamoxifen was administered to label a subset of Keratin 14 (*KRT14*)-expressing progenitor cells with tdTomato. *KRT14* is expressed in stem cells and transient amplifying progenitor cells. This *KRT14*^CreER^:tdTomato label allowed observation of the topography of filiform and fungiform papillae in the form of a papillae map (Fig 7A). Two immediately adjacent papillae (8 and 9) were used as fiduciary points to confirm the correct location before imaging (arrows, Fig 7B). In addition to observing cells in the taste bud, tdTomato^+^ cells were also observed in stem cells and transient amplifying precursor cells surrounding the taste bud (white arrows, Fig 7C). By day 7, a few tdTomato-labeled cells had entered the representative taste bud 6 (tb6; Fig 7C). The two tdTomato-labeled cells inside the taste bud were segmented in Imaris so that fluorescence outside the cells could be removed, which allowed them to be viewed in the z-axis (Fig 7D). By day 11, a new cell had entered the taste bud (white arrowhead, Fig 7E). When considering the morphology of cells in tb6, cell 1 exhibited mature taste cell morphology (characterized by extension of an apical process into the taste pore), whereas cells 2 and 3 exhibited immature taste cell morphology (Fig 7F). By day 13, one precursor cell (between cells 1 and 3) was lost and never matured (Fig 7G); however, the other three cells remained in the taste bud through day 15 (Fig 7H). Most precursor cells had entered the taste bud by 14 days post-tamoxifen injection (Fig 7I). The taste receptor type 1 member 3 (*T1R3*) promoter was used to label Type II taste bud cells with GFP [27]. All three cells in tb6 were *T1R3*^GFP+^ (Fig 7J); however, taste bud 2 (tb2) had both GFP^+^ (white arrow) and GFP^-^ (white arrowhead) tdTomato-labeled cells (Fig 7K).

### Potential problems

**Fig 8:**
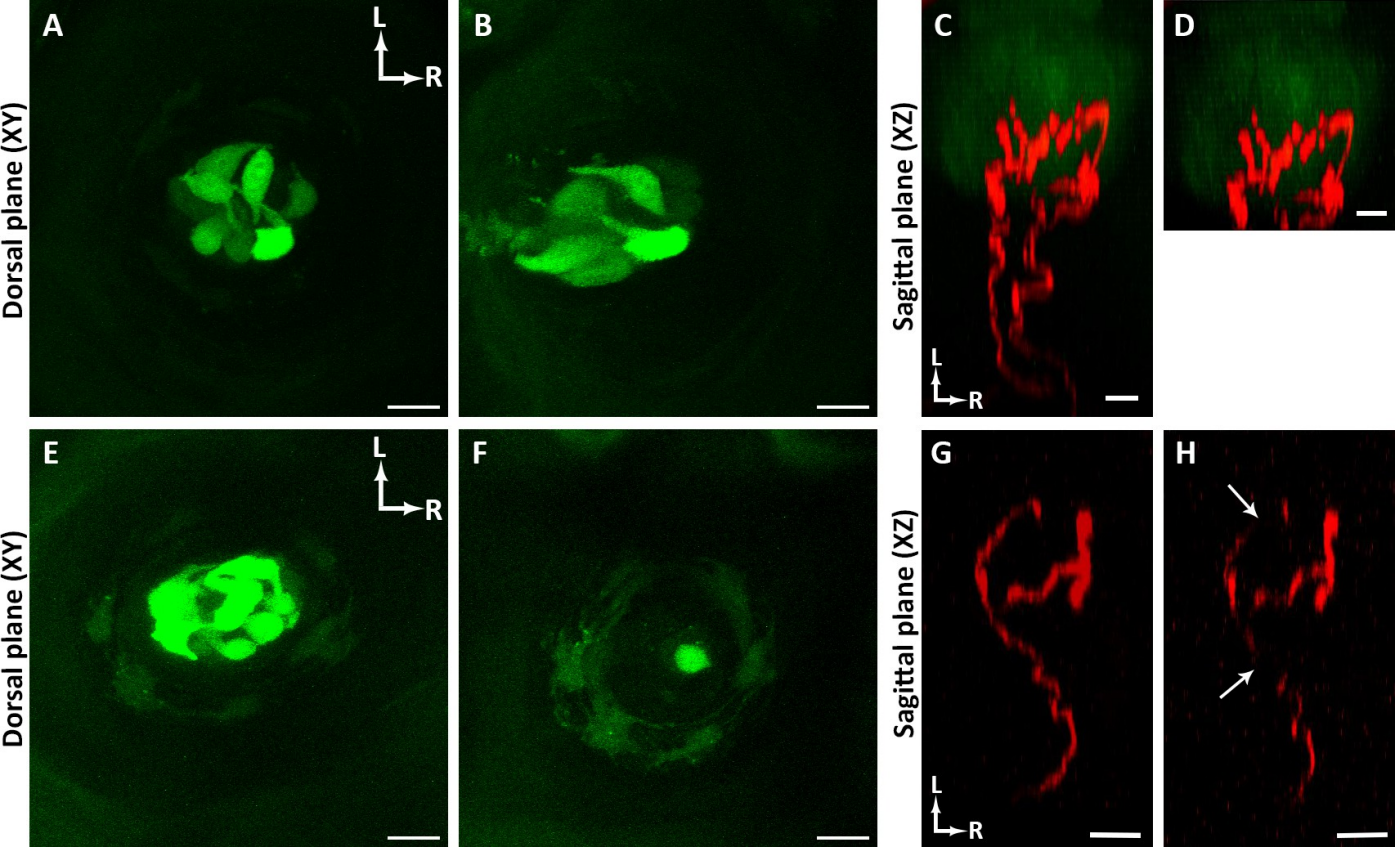
Potential problems. (**A-B**) The same taste bud imaged in a *T1R3*^GFP^ mouse without (**A**) and with (**B**) some tongue movement. If the tongue moves during imaging, then some optical sections are shifted relative to other optical sections. (**C-D**) The same taste bud imaged in a *Sox2*^GFP^:*TrkB*-tdTomato mouse to a sufficient depth to observe axon branching (**C**) and an insufficient depth (**D**) as branching characteristics below the taste bud are absent. (**E-F**) A taste bud from a *T1R3*^GFP^ mouse which after day 16 of imaging (**E**) cells were lost by day 18 (**F**). One possible explanation is insufficient blood flow which can be prevented by inserting a spacer. (**G-H**) Example of an arbor from a *TrkB*-tdTomato mouse imaged with sufficient laser power striking the specimen (**G**) compared with the same arbor imaged with insufficient laser power (**H**). Rostral and lateral directions are indicated by white arrows in A and C. Scale bar for A-F: 10μm; scale bar for G and H: 5μm.

A few issues can arise when performing intravital imaging. If the mouse is too deeply anesthetized, its tongue can jerk, resulting in an image stack with optical sections shifted relative to other optical sections in the z-stack (Fig 8A and 8B). Failing to image arbors to a sufficient depth beneath the taste bud is another common problem (Fig 8C). If optical sections are not acquired until the arbor is no longer visible beneath the taste bud (Fig 8D), then this may result in incomplete information about the arbors innervating the taste bud. Specifically, branching below the taste bud confirms that two arbors are from the same neuron (Fig 8D). Additionally, the ability to accurately orient the arbors in 3D across time for comparison of features (e.g., secondary branches, terminal branches) is reduced if the entire arbor is not acquired at each imaging session. Tissue damage can arise from insufficient blood flow, which can damage the taste bud (19). This may have occurred in a *T1R3*^GFP^ mouse tongue (Fig 8E and 8F). *T1R3*^GFP^ labels all Type II cells in each taste bud [29]. Initially, this taste bud had seven GFP^+^ Type II cells (Fig 8E), but imaging 48 h later revealed that only one cell remained (Fig 8F). This problem can be avoided by imaging with a rigid spacer beneath the cover glass holder. Lastly, problems with the laser power can make it difficult to visualize primary/secondary arbors (insufficient power in Fig 8G, sufficient power in Fig 8H). The arbor in Fig 8H would be difficult to reconstruct using Neurolucida software.

## Discussion

*In vivo* imaging permits the tracking of cell entry, cell loss, cell lifespan, and arbor plasticity in the taste bud. Although fixed tissue and whole mount preparations have been effective in estimating an average cell lifespan and in demonstrating arbor remodeling, *in vivo* imaging of tongue tissue provides more accurate information about the lifespan of individual cells and arbor plasticity [12, 15, 16, 18, 35].

Intravital imaging has major advantages over previously developed methods. It significantly reduces the number of animals needed to conduct a study compared with fixed tissue studies, which require animals to be sacrificed at distinct time points to analyze cell number and/or arbor structure over time. It is the only method that allows the same taste buds to be tracked repeatedly, eliminating sampling variability among taste buds, which remains a confounding variable in fixed tissue and whole mount preparation studies. Additionally, within the taste bud, numerous genetic markers for different taste cell types can be used along with stains and dyes to observe cellular organelles.

However, this method is not without its limitations. A limited region of the anterior tongue surface is visible through the cover glass holder, meaning that imaging other regions would require repeated repositioning of the tongue. As a result, there is not enough time to image all taste buds on the anterior tongue in one imaging session. Additionally, as it is not possible to image the posterior tongue, studies using intravital imaging will be a biased sample consisting primarily of fungiform papillae located on the anterior tip of the tongue. Lastly, there are fewer genetic labels and stains suitable for use with *in vivo* tissue than in fixed tissue. In fact, many stains that work in culture may not penetrate the taste bud *in vivo*, because of the permeability barrier of the taste bud [5]. Therefore, whether intravital imaging can be used in a study depends on the question posed and the labeling required to answer the specific question.

In summary, longitudinal *in vivo* two-photon laser scanning microscopy can be used to repeatedly track the same taste buds across time, allowing direct observation of the rate of cell movement (i.e., cell entry and exit) and arbor plasticity in taste buds. It is also suited for tracking the initial entry of precursor cells into the taste bud, cell differentiation in the taste bud, and cell lifespan from maturity to death. Additionally, chemotherapeutic agents can be administered via oral gavage to observe drug effects on the same taste buds before and after drug delivery [26]. This novel method represents a significant advance toward defining the lifespan of taste cell types and observing arbor plasticity in the taste bud.

## Supporting information

S1 File Step-by-step protocol, also available on protocols.io DOI: dx.doi.org/10.17504/protocols.io.eq2lyjqoelx9/v1. Data supporting Fig 7 are published on Mendeley Data: DOI: 10.17632/nhf6pbzc9d.1.
(PDF)
