# Supplementary material for: Longitudinal imaging of the taste bud in vivo with two-photon laser scanning microscopy
Source: PLoS One. 2024 Dec 13;19(12):e0309366. doi: 10.1371/journal.pone.0309366 (PMC11642993; doi:10.1371/journal.pone.0309366)
Supplement: S1 File — Data supporting Fig 7 are published on Mendeley Data: DOI: 10.17632/nhf6pbzc9d.1. (PDF) [file pone.0309366.s001.pdf]

Aug 14, 2024

Protocol for longitudinal imaging of the taste bud in vivo with two-photon laser scanning microscopy

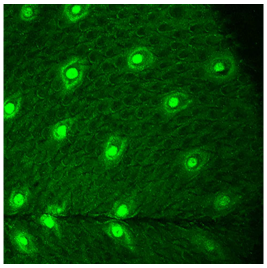

DOI
dx.doi.org/10.17504/protocols.io.eq2lyjqoelx9/v1

Brittany N Walters[1], Zachary D Whiddon[2], Aaron W McGee[1], Robin F Krimm[1]

[1]University of Louisville School of Medicine; [2]University of California San Diego

Brittany N Walters
University of Louisville School of Medicine

OPEN ACCESS

DOI: dx.doi.org/10.17504/protocols.io.eq2lyjqoelx9/v1

Protocol Citation: Brittany N Walters, Zachary D Whiddon, Aaron W McGee, Robin F Krimm 2024. Protocol for longitudinal imaging of the taste bud in vivo with two-photon laser scanning microscopy. **protocols.io**
https://dx.doi.org/10.17504/protocols.io.eq2lyjqoelx9/v1

License: This is an open access protocol distributed under the terms of the **Creative Commons Attribution License**, which permits unrestricted use, distribution, and reproduction in any medium, provided the original author and source are credited

Protocol status: Working
We use this protocol and it's working

Created: December 11, 2023

Last Modified: August 14, 2024

Protocol Integer ID: 92123

Keywords: taste bud, tongue, two-photon laser scanning microscopy, intravital imaging, tissue imaging

Funders Acknowledgement:
NIDCD
Grant ID: DC018878
NIDCD
Grant ID: DC007176
NIDCD
Grant ID: DC019050

Abstract

Here we combined chronic *in vivo* two-photon laser scanning microscopy with genetic sparse labeling of gustatory neurons to repeatedly observe taste cells and arbors (nerve fibers) in taste buds across time. This method expands the investigative possibilities offered by previously developed methods, and we demonstrate a number of these, such as: differentiation of cells and observation of cell turnover, arbor plasticity, and a few stains/dyes that can be used *in vivo* to observe nuclei and organelles in the taste bud. A workflow for reconstructing the composite z-stacks with grayscale data of both cells and arbors using ImageJ, Neurolucida 360, and Neurolucida Explorer software are also provided. Together, the method and software options for analysis presented here demonstrate a novel approach for repeatedly observing taste cells and arbors *in vivo* in the taste bud.

Guidelines

- A comprehensive list of reagents/materials/equipment/mice with corresponding order numbers is available under the 'Materials' section.
- Aim to withdraw and position the mouse tongue in the same orientation beneath the tongue holder at each imaging session.

Materials

A	B	C	D	E
Name of Reagent/Material Equipment/Mice	Company	Catalog Number	Comments/Description	Part ID
Heavy Duty Lab Jack	Thorlabs	L490		
12" x 12" x 3/8" Mini-Series Aluminum Breadboard	Thorlabs	MS12B	Cut to 12" x 6" x 3/8"	
Mini-Post Holder with Swivel Base, 3/4"	Thorlabs	MSH075	Quantity 4	A
4-40 Cap Screw and Hardware Kit for Mini-Series	Thorlabs	HW-KIT5	Kit includes M3 screws and M3 washers as well as hex wrenches that are compatible with the mini series components.	B
Mini-Series Optical Post	Thorlabs	MS2R	Quantity 2	C
Mini-Post Swivel Post Clamp, 360° Continuously Adjustable	Thorlabs	MSWC	Quantity 2	D
Mini Series Posts, 6mm, 1.5"	Thorlabs	MS1.5R	Quantity 1	E
Cage Plate Stops	Thorlabs	ERCPS	Quantity 2	F
Mini-Series Optical Post, Ø6 mm, L = 3"	Thorlabs	MS3R	Weld to nosecone	G
nosecone	custom-design		Dimensions (l × w × h): 1 cm x 1 cm × 1 cm<br>Dimensions (l × w) of two prongs for attachment of tubing: 0.7 cm × 0.4 cm	
4000-2/30 High Performance Rotary Tool Kit	Dremel	F0134000AE		
coverglass holder	custom-design		Dimensions (l × h) of coverglass holder: 3.2 cm × 0.9 cm<br>Dimensions (w) for outer circular portion: 0.9 cm and (w) for post portion: 0.4 cm<br>Dimensions (l × w) for inner circular portion: 0.5 cm × 0.5 cm	
Platinum Coverglass, 24x60 mm, #1	Electron Microscopy Sciences	71887-23		
Diamond Tip, Retractable Scriber	Electron Microscopy Sciences	70036		
stainless steel metal plate	custom-design		Dimensions (l × w × h): 6.3 cm x 1 cm × 1 cm<br>Dimensions (l × w) of cut-out portions on sides of metal plate: 1 cm × 0.4 cm<br>Dimensions (l × w) of the rounded portion of the metal plate: 0.8 cm × 0.3 cm	
silicone tubing			Dimensions: 5/16" inner diameter (ID)	

A	B	C	D	E
Isoflurane	Henry Schien	66794001710		
V-1 Tabletop Laboratory Animal Anesthesia System	VetEquip	901806		
Low-Voltage Animal Temperature Controller W/ Heat Pad & Probe	Physitemp Instruments LLC	TCAT-2 LV		
Ophthalmic Lubricant Ointment	Henry Schien	18582		
Cotton Tipped Applicators				
Plastic Forceps	Fine Science Tools	11700-04		
Objective W "Plan-Apochromat" 40x/1.0 DIC M27	Carl Zeiss	421462-9900-000		
Objective W "N-Achroplan" 10x/0.3 M27	Carl Zeiss	420947-9900-000		
Premium Grade Optical Tissue	Electron Microscopy Sciences	71712-01		
200 Proof Pure Ethanol	Avantor, VWR	V1016		
612/69 nm BrightLine® single-band bandpass filter	SemRock	FF01-612/69-25		
510/84 nm BrightLine® single-band bandpass filter	SemRock	FF01-510/84-25		
Optical Beam Shutter Controller	Sutter Instrument	SC10		
Compact Power and Energy Meter Console, Digital 4" LCD	Thorlabs	PM100D		
Motorized Micromanipulator	Sutter Instrument	MP-285/T		
Photomultiplier Tube	Sutter Instrument	PS-2		
Low Noise Current PreAmplifier	Stanford Research Systems	SR570		
Movable Objective Microscope‰	Sutter Instrument	MOM-5mm		
Fidelity-2: High Power Femtosecond Fiber Laser	Coherent			
Chameleon Ultra: Integrated, Automated and Widely Tunable Ti:Sapphire Oscillator	Coherent			
Neurolucida Explorer	MBF Biosciences	3D vector based image analysis software		
Neurolucida 360	MBF Biosciences	3D vector based image a		

A	B	C	D	E
		nalysis softw are		
Imaris	Bitplane	pixel-based i mage analysi s software		
Tamoxifen	MiliporeSigma	T5648		
Corn oil	MiliporeSigma	C-8267		
Hoechst 33342	Thermofisher Sci entific	H3570	40 µL aliquot of Hoechst 3334 2 was reconstituted in 960 µL of 1x phosphate-buffered salin e (PBS)	
MitoView 405	Biotium	70070-T	50 µL aliquot of MitoView 405 was reconstituted in 75 µL of dimethyl sulfoxide and 400 µL 1x PBS	
Sox2tm2Hoch/J	The Jackson La boratory	#17592		
TrkBCreER mice (Ntrk2tm3.1 (cre/ERT2)Ddg)	The Jackson La boratory	#27214		
Cg-Gt(ROSA)26Sortm14(CAG-tdTomato)Hze/J	The Jackson La boratory	#007914		
B6;129-Tas1r3tm1Csz/J	The Jackson La boratory	#013066		

Safety warnings

Lasers: Class 4
Hazards: flammable, oxidizer, corrosive, irritant, compressed gas (flammable, inert)

Ethics statement

All animals were cared for in accordance with the guidelines set by the U.S. Public Health Service Policy on the Humane Care and Use of Laboratory Animals and the NIH Guidelines for the Care and Use of Laboratory Animals.

Before start

The goal of this protocol is to outline one method for using *in vivo* two-photon laser scanning microscopy to repeatedly image taste buds in the mouse tongue. The first step toward this goal is to construct the imaging platform that the mouse will be placed on for imaging as well as to construct the accessory components (nosecone, metal plate, and cover glass holder) that are required for delivering anesthesia and stabilizing the tongue. (The cover glass holder design was adapted from the holder design presented by Choi et al. 2015.) Subsequent steps in this protocol outline the equipment required to perform *in vivo* two-photon laser scanning microscopy, the process for stabilizing the mouse with anesthesia, and finally, the steps to acquiring papillae maps of the tongue epithelium for repeated imaging of taste buds.

Preparation of Materials

4w

1 Construct the breadboard, nosecone, metal plate, and cover glass holder from the products listed under the 'Materials' section.

1.1 Cut the 12" x 12" x 3/8" Mini-Series Aluminum Breadboard so that its dimensions are 12" x 6" x 3/8". Bolt the breadboard to the Heavy Duty Lab Jack.

1.2 The length × width × height dimensions of the custom-constructed metal plate are 6.3 cm × 1 cm × 1 mm. The length × width dimensions of the inner cut out portion of the metal plate are 1 cm × 0.4 cm, and of the rounded portion of the metal plate are 0.8 cm x 0.3 cm.

1.3 The length × width × height dimensions of the custom-constructed nosecone are 1 cm × 1 mm × 1 cm. Two prongs should also be welded to the top and bottom of the nosecone for delivery of isoflurane and oxygen via silicone tubing. The length × width dimensions of the prongs to which the tubing is attached are 0.65 cm × 0.4 cm.

1.4 Construct the cover glass holder from a thin piece of metal. Use a marker to outline the shape of the cover glass holder on the metal, including the inner rim where the coverslip will be applied. (Dimensions: the entire length of the cover glass holder is 3.2 cm and the height is 0.9 cm. The length × width of the outer circular portion of the cover glass holder is 0.9 cm × 0.9 cm. The length × width of the inner circular portion is: 0.5 cm × 0.5 cm).

1.5 From the Dremel set, first load the EZ Lock Mandrel No 402 onto the rotary tool and proceed by loading the accessory stainless steel wire brush. With these accessories on the rotary tool, manually drill along the outline of the cover glass holder. Change the accessory of the rotary tool to Mandrel No. EZ407SA which will fit the sanding band needed to sand the rough edges of the cover glass holder. (Please refer to the Dremel user manual for a complete guide of operating and safety instructions.)

1.6 Once the cover glass holder has been formed, apply superglue to the inner rim of the cover glass holder and press one, 24x60 mm, #1 platinum cover glass into the glue. Allow the coverslip to dry and adhere to the cover glass holder before using a diamond tip scribe pen to remove the pieces of coverslip that extend from the sides.

1.7 Construct the custom-built imaging platform from the products listed under the 'Materials' section.

1.8 Position the four Mini-Post Holders with Swivel Base, 3/4" (Figure 1, panel A, Part A), onto the breadboard at the four locations indicated: b8 & c10, f8 & g9, f4 & g3, and f2 & g1.

1.9 Secure the Mini-Post Holders to the breadboard using the 4-40 M3 Washers and Cap Screws (Figure 1, panel A, Part B) which are included in the Hardware Kit for Mini-Series. In addition,

secure the metal plate on top of the two Mini-Post Holders.

1.10 Insert two Mini-Series Optical Posts (Figure 1, panel A, Part C), through the Mini-Post Holders at locations b8 and f2 of the breadboard.

1.11 Slide two Mini-Post Swivel Post Clamps, 360° Continuously Adjustable (Figure 1, panel A, Part D), onto the two Mini-Series Optical Posts.

1.12 Insert one Mini Series Post, 6mm, 1.5", into the Mini-Post Swivel Post Clamp (Figure 1, panel A, Part E).

1.13 Attach two Cage Plate Stops (Figure 1, panel A, Part F), onto the Mini Series Post. Use the screws provided with the Cage Plate Stops to secure them to the Mini Series Post.

1.14 Insert one Mini-Series Optical Post L=3" (Figure 1, panel A, Part G) with the nosecone welded to it, into the Mini-Post Swivel Post Clamp.

1.15 A mixture of isoflurane and oxygen from the anesthesia system is delivered via silicone tubing to the nosecone. Slide the silicone tubing over the top and bottom prongs of the nosecone.

2

Turning on equipment

3m

2.1 Open the Matlab ScanImage software associated with the moveable objective microscope (MOM).

2.2 Insert the 612/69 nm single-band bandpass filter (filter signals to better observe tdTomato), and 510/84 nm single-band bandpass filter (filter signals to better observe Green Fluorescent Protein) into the MOM.

2.3 Turn on the Chameleon Ti:Sapphire mode-locked, tunable laser (Coherent) by turning the key from STANDBY to ON. Press the “Open Shutter” button on the laser. Tune the laser to 920 nm. Press “Menu Select” to accept the change in wavelength. Turn on the Fidelity-2 1070-nm laser by turning the key from STANDBY to ON.

2.4 Turn on the Optical Beam Shutter Controller by turning the key to ON and pressing the “Enable” button in the lower right-hand panel. Turn on the Compact Power and Energy Meter Console by pressing the ON button.

2.5 Turn on the Motorized Micromanipulator Stage Controller by turning the switch to ON. Turn on the Photomultiplier tube (PMT) by turning the switch to ON. Leave the individual switches for

both
channels OFF.

2.6 Turn ON the switch to the low-voltage animal temperature controller. Shift ON the power button to the Low Noise Current PreAmplifier.

2.7 Open the valve of the O_2 tank.

3

Preparing mouse for imaging

10m

3.1 Place the mouse in the anesthesia induction chamber. Adjust the dial on the V-1 Tabletop Laboratory Animal Anesthesia System to supply a constant flow of oxygen/isoflurane mixture (5.0% isoflurane) to the mouse.

3.2 Place the mouse in the supine position on the custom-built imaging platform. Adjust the dial on the V-1 Tabletop Laboratory Animal Anesthesia System to supply a constant flow of oxygen/isoflurane mixture (0.8-1.5% isoflurane) for the duration of the imaging session.

3.3 Assess anesthetic depth by checking the pedal withdrawal reflex. Position the nose inside the nosecone. Align the chin of the mouse with the rounded cut-out portion of the metal piece.

3.4 Insert the lubricated rectal temperature probe. Apply veterinary ophthalmic ointment to the eyes using cotton tipped applicators.

3.5 Withdraw the tongue with plastic blunt-ended forceps and wet it with artificial saliva. Position the tip of the tongue on the custom-constructed stainless steel metal plate.

3.6 Lower the cover glass holder until it meets the tip of the tongue. If desired, a fixed spacer can be placed between the cover glass holder and the metal plate to limit the possibility of tissue damage.

4

Acquiring papillae maps

20m

4.1 Position the custom-built imaging platform under the MOM. Insert the 10X 0.3 numerical aperture (NA) W-ACHROPLAN (Zeiss) objective into the MOM. Adjust the z-axis rotary knob on the stage controller to lower the objective until it is roughly 1 mm above the cover glass holder.

4.2 The ScanImage Software will populate multiple dialogue boxes when the program is initialized. In the Main Controls dialogue box, fill in the details of the experiment under basename such as: mouse number, taste bud number, date of imaging experiment.

4.3 Create a new folder on the desktop to store the acquired images. In the Main Controls dialogue box, click “File” -> “Set Save Path”. A file explorer dialogue box will open, and from the panel on the left, select “Desktop”, then select the new folder.

4.4 Turn off the lights and close the black drape in front of the MOM to begin imaging. Shift on the switches for both PMT channels.

4.5 Click “FOCUS” in the Main Controls dialogue box to begin imaging. Press the fine focus button twice on the stage controller for smooth scanning.

4.6 Turn the y-axis rotary knob of the stage controller to the left to move the 10X objective upward until the epithelium of the tongue comes into view. Turn the x-axis rotary knob to the right to scroll to the anterior portion of the tongue.

4.7 When the right half of the tongue has been identified for imaging, use the z-axis rotary knob to scroll through the z-slices.

4.8 In the Movement dialogue box, under stack, click “end” to register the initial z-slice of the z-stack which should be taken at the level of the basal lamina. In the same box, under stack, click “start” to register the final z-slice of the z-stack which should be taken at the level of the epithelium.

4.9 In the Main Controls dialogue box, click “ABORT” -> “GRAB” to acquire the image. Repeat this process using steps 4.5-4.9 to create a papillae map of the left half of the tongue.

4.10 Adjoin the maps at the tongue midline to prevent duplicate imaging of taste buds.

Imaging taste buds using the papillae map

1h

5

5.1 Remove the 10X objective and replace it with a 40X 1.0 NA water immersion objective (Zeiss). If you are using a water immersion objective, then add droplets of distilled water to the top of the coverslip. Use the z-axis rotary knob on the stage controller to lower the objective until it is roughly 1 mm above the cover glass holder.

5.2 Turn off the lights and close the black drape in front of the MOM. Shift on the switches for both PMT channels.

5.3 Click “FOCUS” in the Main Controls dialogue box to begin imaging. Press the fine focus button twice on the stage controller for smooth scanning.

5.4 Move the 40X objective upward in the water column by turning the y-axis rotary knob of the stage controller to the left. Using the x-axis rotary knob, locate one of the taste buds, and use the z-axis rotary knob to scroll through the z-slices of the taste bud.

5.5 In the Movement dialogue box, under stack, click “end” to register the initial z-slice of the z-stack which should be taken at the base of the taste bud. In the same box, under stack, click “start” to register the final z-slice of the z-stack which should be taken when the taste pore of the taste bud comes into view. In the Main Controls dialogue box, click “ABORT” -> “GRAB” to begin imaging.

5.6 It is critical to number the taste buds on the papillae map immediately after it has been imaged. To do so, click the “A” icon text tool in the toolbar. Drag the textbox to the location of one taste bud, enter the text, and press Ctrl+b to add the text.

5.7 Once each taste bud has been assigned a number, select “File” -> “Save As” -> “Tiff”. Select the appropriate folder and click “Save”. Repeat this process for all remaining taste buds imaged. Be sure to adjust the basename after each image is acquired so that it accurately reflects the number assigned to the taste bud to be imaged.

5.8 When finished imaging, shift off the switches for both PMT channels. Open the black drape in front of the MOM, and turn on the lights.

5.9 Use the z-axis rotary knob on the stage controller to raise the 40X objective. Clean the 10X and 40X objectives using a small amount of ethanol sprayed on the Premium Grade Optical Tissue.

Viewing papillae maps using ImageJ software

5m

6 Drag and drop the file of the papillae maps from the file folder to the ImageJ toolbar. Adjust the brightness and contrast of the image by selecting “Image” -> “Adjust” -> “Brightness/Contrast” in the toolbar.

6.1 A B&C panel will appear with options for adjusting the Minimum, Maximum, Brightness & Contrast. Scroll through the z-slices in the image stack and adjust these options until the individual taste buds can be identified.

6.2 Adjoin the maps at the tongue midline to prevent duplicate imaging of taste buds.

Removing mouse

5m

7

7.1 Remove the custom-built imaging platform from beneath the MOM. Raise the cover glass holder. Move the nosecone away from the nose and remove the rectal temperature probe.

7.2 Remove the mouse from the custom-built imaging platform and place a droplet of artificial saliva onto its tongue. Return the mouse to its home cage for recovery. Wait for the mouse to regain mobility.

Turning off equipment

3m

8

8.1 Turn the isoflurane regulator to 0% and close the O_2 tank. Turn the switch on the low-voltage animal temperature controller to OFF.

8.2 Turn off the Chameleon Ti:Sapphire mode-locked, tunable laser by turning the key from ON to STANDBY. Turn off the Fidelity-2 1070-nm laser by turning the key from ON to STANDBY. Turn off the Optical Beam Shutter Controller by turning the key to OFF.

8.3 Press the OFF button on the Compact Power and Energy Meter Console. Flip the switch to OFF on the Motorized Micromanipulator Stage Controller. Flip the switch on the Photomultiplier tube to OFF. Turn the switch on the Low Noise Current PreAmplifier to OFF.

Deinterleaving, Merging, and Colorizing z-stacks using ImageJ software

25m

9

9.1 To view the individual taste bud images, open Fiji ImageJ software (**https://imagej.net/software/fiji/downloads**). Select “File” -> “Open” to view the image.

9.2 The composite stacks that are generated contain grayscale data for both cells and arbors in different channels. Arbor data is stored in odd slices and cell data is stored in even slices. This grayscale composite image can be deinterleaved by selecting “Image” -> “Stacks” -> “Tools” -> “Deinterleave”.

9.3 To pseudo-color both channels, select “Image” -> “Color” -> “Merge Channels”. A dialogue box named Merge Channels will populate. To pseudo-color the cells green and arbors red, select “filename.tif#1” in the dropdown box next to C2 (green) and “filename.tif#2” in the dropdown box next to C1 (red). If it is not already checked, then place a check in the box, “Create Composite”. Click “OK”.

9.4 Adjust the brightness and contrast of the image. To save the image, select “File” -> “Save As” -> “Tiff”. Repeat steps 9.1-9.3 to create pseudo-colored composites for all remaining taste buds.

Importing Colorized Composite z-stack files into Neurolucida 360

1h

10 To view the individual taste bud images, open the Neurolucida 360 software. Select “File” -> “Open” -> “Image Stack”. A dialogue box called Image Scaling should appear. The following parameters should be set: The distance between images (size of voxel in Z) should be set to 1 µm. Distance Type should be set to Physical Distance. Correction Factor for Physical Distance should be set to Water. Place a check in the box next to Override X and Y scaling. X and Y Scaling Applied to Images should be set for X and Y to 0.156 µm/voxel.

10.1 The cells and arbors should import into the program pseudo-colored in the colors previously selected in ImageJ.

10.2 Refer to Ohman and Krimm 2021 for further instructions regarding how Neurolucida 360 can be used to trace arbors.

Troubleshooting issues with repeatedly imaging taste buds

11 If you are having difficulties locating the individual papillae repeatedly, then troubleshooting suggestions are listed below:

11.1 *Cannot identify taste buds.* On each map, identify a unique feature that can help you identify two taste buds. This could be their remarkable proximity to each other relative to other taste buds on the tongue or the particular angle that these two (or three) taste buds sit at on the tongue. If you identify this feature on the map with the 10x objective, then you should be more easily able to navigate to it with the 40x objective.

11.2 *Unsure if the correct taste bud has been identified.* Because the arbor beneath the taste bud is stable, the number / shape of arbors can be used to positively identify a taste bud.

Protocol references

Choi M, Lee WM, Yun SH. Intravital microscopic interrogation of peripheral taste sensation. Scientific reports. 2015; 5: 8661. doi.org/10.1038/srep08661.
